# Chemical Profile and Antimicrobial Activity of the Essential Oils of *Helichrysum arenarium* (L.) Moench. and *Helichrysum italicum* (Roth.) G. Don

**DOI:** 10.3390/plants11070951

**Published:** 2022-03-31

**Authors:** Valtcho D. Zheljazkov, Ivanka Semerdjieva, Elina Yankova-Tsvetkova, Tess Astatkie, Stanko Stanev, Ivayla Dincheva, Miroslava Kačániová

**Affiliations:** 1 Crop and Soil Science Department, Oregon State University, 3050 SW Campus Way, 109 Crop Science Building, Corvallis, OR 97331, USA; 2 Department of Botany and agrometeorology, Agricultural University, Mendeleev 12, 4000 Plovdiv, Bulgaria; v_semerdjieva@abv.bg; 3 Department of Plant and Fungal Diversity, Division of Flora and Vegetation, Institute of Biodiversity and Ecosystem Research, BAS, 2, Gagarin Str., 1113 Sofia, Bulgaria; e_jankova@abv.bg; 4 Faculty of Agriculture, Dalhousie University, P.O. Box 550, Truro, NS B2N 5E3, Canada; astatkie@dal.ca; 5 Institute of Roses, Essential and Medical Plants, Agricultural Academy, bul. “Osvobozhdenie” 49, 6100 Kazanlak, Bulgaria; sdstanev@abv.bg; 6 Department of Agrobiotechnologies, AgroBioInstitute, Agricultural Academy, 8 Dragan Tsankov blvd., 1164 Sofia, Bulgaria; ivadincheva@yahoo.com; 7 Institute of Horticulture, Faculty of Horticulture and Landscape Engineering, Slovak University of Agriculture, Tr. A. Hlinku 2, 94976 Nitra, Slovakia; kacaniova.miroslava@gmail.com; 8 Department of Bioenergetics and Food Analysis, Institute of Food Technology and Nutrition, University of Rzeszow, 35-601 Rzeszow, Poland

**Keywords:** Bulgaria, Bosnia, Corsica, France, essential oil, Helichrysum, protected species, *α*-pinene, sabinene, neryl acetate

## Abstract

This study compared the essential oils (EO) composition of *Helichrysum arenarium* (Bulgarian populations) with that of the cultivated species *H. italicum*. The EO composition of *H. arenarium* and *H. italicum* were analyzed via gas chromatography. In general, 75 components were identified in *H. arenarium* EO and 79 in *H. italicum* EO. The predominant constituents in *H. arenarium* EO were *α*-pinene (34.64–44.35%) and sabinene (10.63–11.1%), which affirmed the examined population as a new chemical type. Overall, the main EO constituents of *H. italicum* originating in France, Bosnia and Corsica were neryl acetate (4.04–14.87%) and *β*-himachalene (9.9–10.99%). However, the EOs profile of *H. italicum* introduced from the above three countries differed to some extent. D-limonene (5.23%), italicene, *α*-guaiene and neryl acetate (14.87%) predominated in the *H. italicum* introduced from France, while *α*-pinene (13.74%), δ-cadinene (5.51%), *α*-cadinene (3.3%), *β*-caryophyllene (3.65%) and *α*-calacorene (1.63%) predominated in plants introduced from Bosnia. The EOs of the plants introduced from France and Corsica had similar chemical composition and antimicrobiological activity.

## 1. Introduction

Plants species belonging to *Helichrysum* Mill. genus (Asteraceae) have long been known for their healing properties, and preparations based on *Helichrysum* species have been and continue to be used around the world [1]. The pharmaceutical, cosmetic and perfume industries have taken a strong interest in *Helichrysum* species because of the specific essential oil (EO) aroma and composition [2]. Extracts from *Helichrysum* species possess a wide range of pharmacological activities such as antioxidant, antimicrobial, antiatherosclerotic, antiproliferative, antidiabetic, neuroprotective and antiinflammatory activities [3,4,5,6]. There are 16 species in the *Helichrysum* genus spread across Europe [7]. Two species, *H. arenarium* (L.) Moench. and *H. plicatum* DC. [8], are naturally occurring in Bulgaria, while *H. italicum* (Roth) G. Don. is an introduced cultivated species in this country. Products derived from *H. italicum* are widely used in the traditional medicine, cosmetics and the food industry and are particularly popular in the Mediterranean countries [9,10]. In recent years, there has been increasing interest in products from *H. italicum*. As a result, the species has been commercially cultivated in France [11], Portugal [12], Bosnia and Herzegovina [2,13], Italy [14,15], Serbia [10] and recently in Bulgaria. There has been significant interest in the phytochemical composition and pharmacological activity of *H. italicum* during the last decade and a half [13,15,16,17,18].

*Helichrysum arenarium* has a long tradition as a medicinal plant in the European ethnomedicine [19]. Medicines based on *Helichrysi* flos were enlisted in the State Pharmacopoeia of the USSR [20], Pharmacopoeia Helvetica [21] the Polish Pharmacopoeia [22], as well as in a herbal monograph on *H. arenarium* [23]. Because of its healing properties, *H. arenarium* has been collected from its natural populations; hence, wild collection has the potential to disturb stable populations of this species. In some European and Asian countries, such as Sweden, Poland, Kazakhstan and Serbia, the species is protected and cultivated [24,25,26]. In Bulgaria, *H. arenarium* is protected according to the Biodiversity Act, included in Annex 4 of this act and in the List of Species of Medicinal Plants under special regimen of conservation [27,28].

Research studies related to its phytochemical composition have focused mainly on the content of phenols and flavonoids [29,30,31,32,33,34,35,36,37,38,39,40,41,42,43,44,45,46,47,48,49,50,51,52] (Table 1). This is not accidental because phenolic compounds, including flavonoids (like in *Helichrysi* flos), used in traditional medicine (biological source *H. arenarium*) have been demonstrated to have cholagogue, choleretic, hepatoprotective and inhibitory effects on tumor necrosis; in addition, these compounds are used to make a detoxifying herbal drug [19,30,40]. Studies on EO composition of *H. arenarium* are limited and the existing data for EO composition diverge widely [4,30,31,32,33,35] (Table 1). For example, in a study of the Hungarian population, the predominant EO constituents were linalool, carvacrol, anethole, anisaldehyde and thymol [31,32]; in Serbia, major EO constituents included diepi-*α*-cedrene, *α*-ylangene, cyclosativene and limonene [4]; in Iran, spathulenol, *β*-pinene, limonene, alpha-cadinol and borneol were observed [43,45]. The latter authors concluded that the observed differences in the EO composition were due to the different geographical habitats of the species [4,31,32,33,43,45]. So far, phytochemical studies of the Bulgarian wild populations of *H. arenarium* have not been conducted. To preserve the natural population of the species, it may need to be cultivated ex situ through its development as a cultivated crop. Phytochemical studies are necessary for the selection of accessions possessing high content and desirable composition of the EO. Overall, information on the EO composition of *H. italicum* cultivated in Bulgaria is limited.

Previous research showed that *H. italicum* EO exhibited antioxidant, antimicrobial, antiviral, anti-inflammatory, and antiproliferative activity [13]. *H. italicum* showed low or no activity against tested bacteria. However, for all Gram-negative bacteria (*E. coli, P. aeruginosa, Salmonella typhimurium, S. enteritidis, K. aerogenes* and *P. hauseri*) minimum inhibitory concentration (MIC) and minimum bactericidal concentration (MBC) values were higher than 454.5 µL/mL EO. For the Gram-positive bacteria (*B. cereus*, *L. monocytogenes*, *R. equi*, and *S. epidermidis*) MIC and MBC values was 454.5 µL/mL, while for other (*B. spizizenii, E. faecalis, L. innocua, L. ivanovii*, and *S. aureus*) MIC and MBC values were higher than 454.5 µL/mL of EO [10].

The present study analyzed the EO composition of the Bulgarian population of *H. arenarium* and compared it with the EO of *H. italicum*, which already has an established international market. The working hypothesis was that EO composition of *H. italicum* and *H. arenarium* would be similar.

## 2. Results

### 2.1. Qualitative Composition of the Essential Oil (EO)

#### 2.1.1. *Helichrysum arenarium*

**Figure 1:**
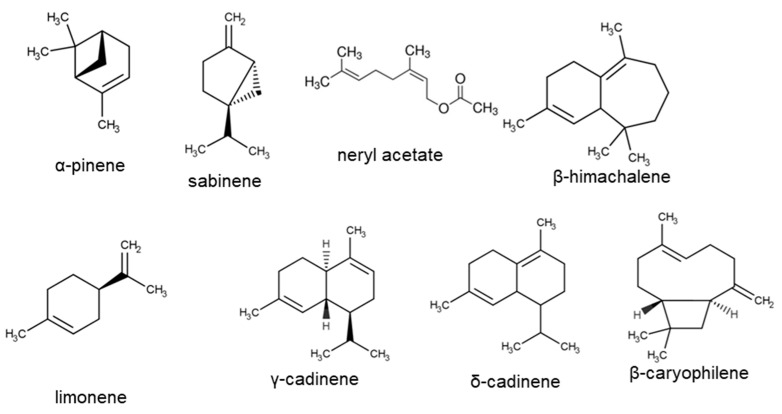
Structural formulas of the main compounds of *Helichrysum arenarium* and *H. italicum*.

The analysis of variance (ANOVA) results that identified significant (bold) and nonsignificant differences among the mean constituents of *H. arenarium* are shown in Table 2. Data from EO analysis of *H. arenarium* are presented in a Supplementary Table S1. Overall, 75 EO constituents were identified and grouped into the following classes: monoterpenes, sesquiterpenes, diterpenoids and long-chain alkane, in total 90.82–94.4% of the total oil. The monoterpenes predominated in the three tested samples (65.72–73.99%) (Table 3): *α*-Pinene (34.64–44.35%) and sabinene (10.63–11.1%) were predominant in the three samples and *β*-pinene, trans-verbenol and D-limonene were observed in similar quantity (Table 3) (Figure 1). With respect to the sesquiterpenes in *H. arenarium* EO, (16.08–19.41%), germacrene D (3.56–4.86%) and *β*-gurjunene (3.61%) were predominant (Table 3). The concentrations of sabinene, D-limonene, trans-verbenol, n-tetradecane and *β*-gurjunene in *H. arenarium* EO were not significantly different between the three locations; the overall means of these compounds are shown in Table 4.

#### 2.1.2. Helichrysum italicum

The ANOVA results that identified significant (bold) and nonsignificant differences among the mean constituents of *H. italicum* are shown in Table 5. The chemical composition of EO of *H. italicum* is presented in Supplementary Table S2, Table 6 and Table 7. The Gas chromatography-mass spectrometry (HS/GC-MS) analyses showed 79 EO constituents (Supplementary Table S2). Generally, sesquiterpenes were predominant in the EO from all three locations: 45.23% (France), 47.9% (Corsica) and 54.8% (Bosnia). *β*-Himachalene was found to be present in the three analyzed samples in the range of 9.9% to 10.9% (Table 6), although EO of each of those samples possessed a specific profile. For example, italicene and *α*-guaiene were present in higher quantity in the plants introduced from Corsica and France (Table 6). The EO of plants introduced from Bosnia was characterized by higher content of *δ*-cadinene (5.51%), *α*-cadinene (3.3%), *β*-caryophyllene (3.65%) and *α*-calacorene (1.63%) compared with the other samples (Supplementary Table S2). The structural formulas of some of the main compounds are presented on Figure 1.

Monoterpenes represented the second major class of the *H. italicum* EO. In the EO of plants introduced from France, monoterpenes were observed in the greatest quantity (37.3%), with D-limonene (5.23%) and neryl acetate (14.87%) being the predominant monoterpenes (Table 6). The main EO constituents of plants introduced from Bosnia were *α*-pinene (13.74%) and *p*-cymen-7-ol acetate (5.27%) (Table 6).

#### 2.1.3. Antimicrobial Activity of the *H. italicum* EO

The *H. italicum* EO of plants introduced from Bosnia, Corsica, and France were tested for antimicrobial activity against nine microorganisms using the disc diffusion method. Antimicrobial activity of different microorganisms and location ranged from 2.33 to 14.67 mm. Overall, the EO of *H. italicum* from all locations was more effective against *S. aureus* and ranged between 9.33 and 14.67 mm (Table 8). Moderate antimicrobial effect was found against *C. krusei* and *C. tropicalis*. The lowest antimicrobial activity was found against *Y. eneterocolitca*. In general, the tested EOs were more effective against Gram-positive bacteria.

## 3. Discussion

### 3.1. Helichrysum arenarium

Results of the present study on *H. arenarium* show that *α*-pinene (34.64–44.35%), sabinene (10.63–11.1%), germacrene D (3.56–4.86%), *β*-gurjunene (3.61%), *β*-pinene, trans-verbenol and D-limonene were the predominant constituents of *H. arenarium* EO. Unlike in previously published data on the species, the results from this study affirmed a new chemical type (chemotype) of *H. arenarium* in Bulgaria.

In Bulgaria, *H. arenarium* grows on sandy and coastal habitats at up to 500 m above sea level: the Black Sea coast, the Danube Plain (central part), north-eastern Bulgaria and south-eastern Bulgaria [8]. Previously, Czinner et al. [31] analyzed steam-distilled EO of *H. arenarium* plants collected in the Caucasus region and established that the largest group of compounds was the aliphatic acids (34.6%), among which were dodecanoic acid (11.9%) and decanoic acid (9.8%), followed by ester methyl palmitate (28.5%) and further aromatic compounds (10.2%) such as carvacrol and anethole (3.6 and 3.2%, respectively). On the other hand, Lemberkovics et al. [32], using the same analytical approach, reported that the predominant compound in the EOs of Polish and Hungarian commercial samples was methyl palmitate (21.7–28.5%), while caprinic acid (19.8%) was the main EO constituent in a cultivated plant sample from Hungary. These discrepancies in chemical profiles could be a consequence of different environmental factors, such as isolation, soil type, precipitation, etc. Furthermore, Judzentiene and Butkiene [33] reported chemical profiles of *H. arenarium* EOs from inflorescences and leaves of yellow and orange flowering plants. Apparently, the EO from inflorescences of both types of plants, yellow and orange, had two dominant constituents, *β*-caryophyllene and heneicosane, followed by *α*-copaene (9–25.6%, 3–32.1% and 1.5–7.2%, respectively). One of the main constituents in the EOs extracted from leaf in both plant types with yellow and orange inflorescences, excluding *β*-caryophyllene, was δ-cadinene (9.8–22.3% and 6.6–11.8%, respectively). Other EO constituents included 1,8-cineole, *α*-copaene, (E)-*β*-ionone, *γ*-cadinene, selina-3,7(11)-diene, epi-*α*-cadinol, *α*-cadinol, octadecane, isophytol and tricosane [33].

Analyses of the composition of the EO from Central European samples were conducted by several authors [31,33,35]. These analyses also reported differences between EO obtained from different geographic locations. Samples from the Caucasus region analyzed by Czinner et al. [31] showed the presence of 1.5% of *β*-asarone, which was not found in the samples from Central Europe.

### 3.2. Helichrysum italicum

*Helichrysum italicum* is a thermophilic plant species, which is among the most frequently studied species [13]. The plants introduced from Bosnia, France and Corsica had differing and specific composition of EO. For example, the basic constituents in EO of the plants introduced from Bosnia were *α*-pinene, *β*-caryophyllene, p-cymen-7-ol acetate and *β*-himachalene (Table 5), while the predominant constituents in EO of the plants introduced from France and Corsica were D-limonene, neryl acetate, nerol, italicene, *α*-guaiene, *γ*-eudesmol, 2-methyl butyl-2-methyl butyr and *β*-himachalene. The established differences could be due to the fact that *H. italicum* is characterized by high polymorphism, spontaneous hybridization and variations in EO composition [3,13]. Review of the literature provided evidence that variation in the *H. italicum* EO composition could be due to numerous factors, such as geographical origin, ecological factors, geographical features of the habitat, the sampled part of the plant, the relevant stage of growth and the extraction methods [10,47,53,54]. Depending on the geographical origin, some researchers reported several chemotypes of *H. italicum*. For example, based on literature data, Ninčević et al. [13] named the following chemotypes for *H. italicum*: (1) EO from Corsica (neryl acetate, neryl propionate, aliphatic ketones and *β*-diketones); (2) EO from Serbia (*α*-pinene, then y-curcumene, *β*-selinene, neryl acetate and *β*-caryophyllene); (3) EO from the Adriatic Coast (*α*-curcumene or *γ*-curcumene or *α*-pinene, neryl acetate); (4) EO from Greece (geraniol, geranil acetat and nerolidol); and (5) EO from Tuscana (*α*-pinen, or neryl acetate, *β*-selinene, *β*-cariophylene and *α*-selinene).

Another classification of the chemotypes of *H. italicum* was generated by Aćimović et al. [10]. Depending on the main constituents, the authors identified 10 chemotypes of *H. italicum*, namely (1) high neryl acetate chemotype (50.5–83.4%), (2) moderate neryl acetate chemotype (19.5–48%), (3) neryl acetate + ar-curcumene (3.9–20.3% and 0.8–14.5%, respectively), (4) ar-curcumene + *γ*-curcumene (17.9–28.6% and 12–22%, respectively), (5) *γ*-curcumene (13.6–27.7%), (6) high *α*-pinene chemotype (25.2–53.5%), (7) moderate *α*-pinene (5.6–20%), (8) juniper camphor (25.3–45.1%), (9) *β*-selinene (11.6–38%) and (10) italidiones chemotype [10].

The samples examined in this study showed different EO profiles and could not be assigned to any of the above-mentioned chemotypes of *H. italicum*. The predominant constituents in EO of plants introduced from Bosnia were *α*-pinene (13.74%), δ-cadinene (5,51%), *α*-cadinene (3.3%), *β*-himachalene (9.9%) and *β*-caryophyllene (3.65%). The EOs of the plant samples introduced from France and Corsica had similar profiles; these contained neryl acetate (12.37–14.87%), *β*-himachalene (9.9–10.99%) and D-limonene (5.23–4.94%).

### 3.3. Comparing the EOs between H. arenarium and H. italicum

The species from the genus *Helichrysum* are widely used in traditional medicine worldwide and they are known as everlasting flowers [1]. As noted in the introduction, some *Helichrysum* species are widespread and others are cultivated in the Mediterranean, Iberian Peninsula and Eastern Europe [13,19,55]. The EO composition of *H. italicum* has been studied widely, while *H. arenarium* has been studied mainly for the content of phenols and flavonoids (Table 1). Data comparing the composition of the EO between the two species have not been published. The samples of *H. arenarium* collected and analyzed in this study contain a specific EO profile that differs from the EO composition of *H. italicum*. Monoterpenes (*α*-pinene, sabinene) were the predominant class of compounds in the EO of *H. arenarium*, while the EO of *H. italicum* was dominated by the class of sesquiterpenes (neryl acetate and *β*-himachalene). However, the EO samples of *H. italicum* from three regions differed from each other to some extent. D-limonene (5.23%), italicene, *α*-guaiene and neryl acetate (14.87%) predominated in the *H. italicum* plants introduced from France, while the *H. italicum* plants introduced from Bosnia had predominantly *α*-pinene (13.74%) and *δ*-cadinene (5, 51%). This difference in the EO composition between *H. arenarium* and *H. italicum* refutes our working hypothesis.

### 3.4. Antimicrobial Activity of the H. italicum EO

Djihane et al. [56] studied antimicrobial activity of *H. italicum* EO against Gram-positive bacteria (G^+^), Gram-negative (G^−^) bacteria and fungi. Gram positive bacteria were more sensitive to the presence of EO than G^−^ bacteria or fungi. The results from this study were similar with our results.

In the current study, fungi were the most resistant class of microorganisms to the presence of the EO. Oliva et al. [57] tested *H. italicum* EO against methicillin-sensitive *Staphylococcus aureus* (ATCC 29213), *Escherichia coli* (ATCC 25922), *Candida albicans* (ATCC 14053) and the clinical strains of methicillin-resistant *S. aureus*, carbapenem-resistant *Klebsiella pneumoniae*, carbapenem-resistant *Acinetobacter baumannii* and carbapenem-resistant *Pseudomonas aeruginosa*. Interestingly, fungicidal/bactericidal potency against *C. albicans* and carbapenem-resistant *A. baumannii* was revealed at a concentration of 5% *v*/*v*. Staver et al. [58] conducted antimicrobial assays and showed that EO had weak to moderate antimicrobial potential with *S. aureus* and *S. epidermidis* as the most sensitive bacterial strains. In the study of Dzamic et al. [59], the most sensitive bacteria to *H. italicum* EO were *Bacillus cereus* and *Salmonella typhimurium*, while the most sensitive fungus was yeast, *Candida albicans*. Similar to our study, Mollova et al. [60] showed *H. italicum* EO from France had more pronounced antimicrobial activity against the G^+^ bacteria *Staphylococcus aureus*, *Bacillus subtilis*, and the fungus *Aspergillus brasiliensis*, as well as a stronger antioxidant potential compared with the other EOs. The obtained results from this study are in good agreement with the findings of Cantore et al. [61] who reported that G^+^ bacteria are more sensitive to plant EOs than G^−^ bacteria. Mesic et al. [62] reported that immortelle essential oil inhibited only Gram-positive bacteria and possessed antifungal effects.

With respect to the antibacterial properties of *H. italicum* EO and its related constituents, Rossi et al. [63] demonstrated that the EO obtained from endemic plants of Corsica was more effective against the G^+^ bacterium *S. aureus* than against the G^−^ strains *E. coli*, *Enterobacter aerogenes* and *P. aeruginosa*. In our study, the most resistant microorganisms tested were G^−^ bacteria. It is commonly known that G^−^ bacteria are less susceptible to EO than G^+^ bacteria, and this is directly connected to the bacterial cell wall structure. In G^−^ bacteria, the cell wall is a complex envelope constituted by the cytoplasmic membrane, the periplasm and the outer membrane. Results reported in Cantore et al. [61] and Rossi et al. [63] are consistent with those in our study. Antimicrobial activity of *H. italicum* EO from Algeria with *α*-cedrene, *α*-curcumene and geranyl acetate as dominant compounds assayed by disk diffusion method inhibited growth of *S. aureus, M. luteus, E. cereus, B. cereus, S. epidermidis, B. subtilis, P. aeruginosa, E. faecalis* and *P. mirabilis*, but did not affect *E. coli, K. pneumonia* and *L. monocytogenes*. In addition, yeasts (*C. albicans* and *S. cervisae*), as well as fungi (*F. solani, A. niger, A. alternata* and *A. rabiei*), were also inhibited by *H. italicum* EO [64].

## 4. Materials and Methods

### 4.1. Plant Material

**Figure 2:**
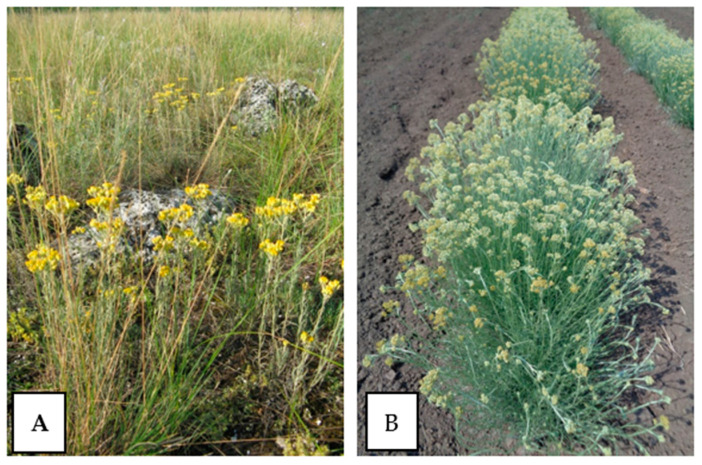
Wild population of *Helichrysum arenarium* of population Pobitite kamani, near Varna town (**A**), cultivated *Helichrysum italicum* (**B**).

The materials utilized in this study were aerial parts in full flowering. The plant materials of *H. arenarium* were collected from three locations (numbered 689; 691; 699) of population Pobitite kamani, near Varna town (Figure 2A) (43.228196 N; 27.705116 E; 114 masl) with an official permit (#790/19.04.2019 of MOCB). The collected samples were air-dried at room temperature until a constant weight. Voucher specimens of *H. arenarium* were deposited at the Herbarium of the Agricultural University, Plovdiv, Bulgaria (SOA) [65].

Samples of *H. italicum* introduced from Bosnia, France and Corsica and grown side by side in Bulgaria were obtained from experimental fields at the Institute of Roses, Essential and Medical Plants in Kazanlak, Bulgaria (Figure 2B). These plantations were established via vegetative propagation/rooting of fresh green cuttings prepared from the original imported plants. The plants originating from Bosnia and Herzegovina and Corsica were imported to Bulgaria as seedlings in trays, while the plants from France were imported and grown from collected seeds.

### 4.2. Essential Oil (EO) Extraction

The EO of all samples was extracted via hydrodistillation in a 2 L Clevenger-type apparatus (Laborbio Ltd. Sofia, Bulgaria, laborbio.com, accessed on 12 June 2021). Since the samples from *H. arenarium* were much smaller, the EO of *H. arenarium* was isolated from 45 g of flowering aerial parts of each accession by hydrodistillation in a Clevenger-type distillation unit for 2 h plus in 0.8 L of water. The duration of the hydrodistillation was 2 h and all samples were extracted in two replicates.

Samples from the introduced and grown in Bulgaria materials of *H. italicum* were 1000 g of fresh aboveground plant material in the full flowering stage. In addition, to obtain larger EO samples for biological activity testing, steam distillation was performed in 5 L metal cylindrical containers using 1.5 L of water under the grate on which the raw material was placed. The steam distillation time was 1.5 h. The EO extraction was done at the Institute of Roses, Essential and Medical Plants in Kazanlak, Bulgaria, and each extraction was performed in two replicates. After isolation of each subsample, EO volume and weight were measured, and the EO samples were stored in a freezer at 4 °C for further analyses.

### 4.3. Gas Chromatography (GC) Flame Ionization Detection (FID) and Gas Chromatography–Mass Spectroscopy (MS) Analyses of the Essential Oils (EO)

The chemical profiles of the *H. italicum* and *H. arenarium* EO, in two replications, were determined by GC-FID and GC/MS techniques using a 7890A gas chromatograph (Agilent Technologies Inc., Santa Clara, CA, USA), according to the methods described in our previous study [66]. The GC-MS analysis was performed on a 7890A gas chromatograph (Agilent Technologies Inc., Santa Clara, CA, USA) coupled directly to an Agilent mass selective detector (MSD-5975C). The system was equipped with a HP-5ms fused silica capillary column (5% phenyl 95% dimethylpolysiloxane, 30 m × 0.32 mm i.d., film thickness 0.25 μm, Agilent Technologies, USA). The oven temperature was programmed from 40 °C to 300 °C at a rate of 5 °C/min, and held for 10 min. The temperatures of the injector, the MS quadrupole and the ion source were 250 °C, 150 °C and 230 °C, respectively. The MSD transfer line was maintained at 270 °C.

All mass spectra were acquired in the EI mode (scan range of *m*/*z* 50–500 at 1 s/decade; ionization energy of 70 eV). Split ratio was 1:10. The constituents present in the EO samples were identified by comparing their linear retention indices, estimated using a mixture of a homologous series of aliphatic hydrocarbons from C8 to C40 and MS fragmentation patterns with those from an Adams mass spectra library and NIST′08 (National Institute of Standards and Technology).

The GC analysis was performed on an Agilent GC-7890A gas chromatograph (Agilent Technologies, USA) equipped with a flame ionization detector (FID) and HP-5 silica fused capillary column (30 m length × 0.32 mm i.d. × 0.25 µm film thickness) under the same conditions as described above. The FID temperature was maintained at 280 °C for the oil analyses. The relative composition of the investigated samples was calculated on the basis of the GC-FID peak areas (measured using the HP-5 ms column) without using a correction factor.

The GC-FID analysis of the EO was performed with a gas chromatograph 7890A gas chromatograph (Agilent Technologies Inc., Santa Clara, CA, USA) coupled to a flame ionization detector (FID) and HP-5 silica fused capillary column (30 m length × 0.32 mm i.d. × 0.25 µm film thickness). The oven temperature was programmed as mentioned above. The detector and injector temperatures were 280 °C and 220 °C, respectively. The carrier gas was helium at a flow rate of 1 mL/ min. Essential oil samples (1 μL) were injected using the split mode. The percentage composition of EO samples was calculated using the peak normalization method.

### 4.4. Method for Testing Antimicrobial Activity

The EO of *H. italicum* (plant material originated in Bosnia, France and Corsica) was tested against nine microorganisms with an agar disc diffusion method according to in our previous study [66]. In this study, 0.1 mL of microbial suspension was spread on the Mueller Hinton Agar (MHA, Oxoid, UK) for bacteria and Sabouraud Dextrose agar (SDA, Oxoid, UK) for yeasts. Six mm diameter filter paper discs were used for testing. The filter paper was impregnated with 15 μL of EO and placed on MHA, SDA, respectively, with a microbial inoculum. The MHA was maintained at 4 °C for 2 h and then at 37 °C for 24 h and SDA was maintained at 4 °C for 2 h and then at 25 °C for 24 h. After a 24 h incubation period, the diameter of the inhibition zones was measured (in mm). Chloramphenicol (30 µg, Oxoid, UK) and fluconazole (25 µg, Oxoid, UK) served as positive antimicrobial controls. Antimicrobial activity was measured in triplicate.

#### Microorganisms

Nine strains of microorganisms were used to determine antimicrobial activity of the EOs, including three Gram-positive bacteria (SA-*Staphylococcus aureus* subs. *aureus* CCM 4223, EF-*Enterococcus faecalis* CCM 4224, SP-*Streptococcus pneumonia* CCM 4501), Gram-negative bacteria (PA-*Pseudomonas aeroginosa* CCM 1959, YE-*Yersinia enterocolitica* CCM 5671, SE-*Salmonella enterica* subsp. *enterica* CCM 3807), and yeasts (CA-*Candida albicans* CCM 8186, CK-*C. krusei* CCM 8271, CT-*C. tropicalis* CCM 8223 (CT)). The microorganisms were obtained from the Czech Collection of Microorganisms (Brno, Czech Republic).

### 4.5. Statistical Analyses of the Data

One-way analysis of variance was conducted to determine the effect of (1) collection location of *H. arenarium* on the concentration (%) of *α*-pinene, sabinene, *β*-pinene, D-limonene, trans-verbenol, 1-terpinen-4-ol, n-tetradecane, *β*-gurjunene, germacrene D, germacra-4(15),5,10(14)-trien-1, monoterpenes, sesquiterpenes, long-chain alkane and diterpenoids, and (2) country of origin of *H. italicum* (Bosnia, France and Corsica) on the concentration (%) of *α*-pinene, D-limonene, 2-methyl butyl-2-methyl butyrate, isoamyl tiglate, 1-terpinen-4-ol, nerol, neryl acetate, *α*-copaene, italicene, *α*-cis-bergamotene, *β*-caryophyllene, *p*-cymen-7-ol acetate, *α*-guaiene, *γ*-curcumene, *β*-himachalene, *β*-curcumene, germacrene D-4-ol, *γ*-eudesmol, tau.-muurolol, *β*-eudesmol, monoterpenes, sesquiterpenes, ester and long-chain alkane.

One-way analysis of variance was also conducted to determine if there were significant differences among the three locations where *H. italicum* was collected in terms of nine antimicrobial activities (SA, EF, SP, PA, YE, SE, CA, CK and CT).

For each response variable, the validity of model assumptions was verified by examining the residuals as described in Montgomery [67]. When the effect was either marginally significant (0.05 < *p*-value < 0.1) or significant (*p*-value < 0.05), multiple means comparison was completed using Fisher’s LSD at the 5% level of significance, and letter groupings were generated. The analysis was completed using the GLM Procedure of SAS [68].

## 5. Conclusions

This study assessed the chemical profile of *Helichrysum arenarium* (Bulgarian populations) with that of the cultivated species *H. italicum* introduced from three different countries and grown side by side in Bulgaria. The main components in *H. arenarium* EO were *α*-pinene (34.64–44.35%) and sabinene (10.63–11.1%), indicating a possible new chemotype not previously reported in the literature. The chemical profile of *H. italicum* EO originating in France, Bosnia and Corsica were neryl acetate (4.04–14.87%) and *β*-himachalene (9.9–10.99%); however, there were differences between the EO from plants introduced from the above countries. The *H. italicum* EO plants originating in France, Bosnia and Corsica were evaluated for antimicrobial activity and it was revealed that the EO of plants from France and Corsica had similar composition and antimicrobial activity.

## Figures and Tables

**Table 1:** Literature data of phytochemical research on *Helichrysum arenarium*.

Reference	Main Compounds	Country
Rančić et al. [4]	diepi-*α*-cedrene (17.9%), *α*-ylangene (13.9%), cyclosativene (11.9%), limonene (11.4%)	Serbia
Mao et al. [5]	narirutin, naringin, eriodictyol, luteolin, galuteolin, astragalin, kaempferol	China
Smirnova and Pervykh [29]	flavonoids-astragalin, luteolin, kaempferol	Russian Federation
Czinner et al. [30]	phenolic compound	Hungary
Czinner et al. [31]	linalool (1.7%), anethole (3.2%), carvacrol (3.6%), *α*-muurolol (1.3%), 1.5% of *β*-asarone	Hungary
Lemberkovics et al. [32]	linalool, alpha-terpineol, carvone monoterpenes; anethole, anisaldehyde, thymol, carvacrol, eugenol, beta-asarone, butylhydroxyanisole aromatic components; alpha-humulene, beta-caryophyllene, gamma-muurolene, delta-cadinene, copaene, alpha-gurjunene, caryophyllenol, delta-cadinol and globulol sesquiterpenes, caprylic acid, pelargonic, caprinic, lauric acids, methyl palmitate	Hungary
Judzentiene and Butkiene [33]	*β*-caryophyllene; *δ*-cadinene; octadecane; heneicosane	Lithuania
Bryksa-Godzisz et al. [34]	phenolic compounds	Poland
Radušienė and Judžentienė [35]	1.8-cineole (2.3–7%); *α*-copaene (2.2–3.6%); trans-caryophyllene (4.4–8.8%); epi-a-cadinol (2–4%); m/z-149 (phthalide)(0.6–5.6%); heneicosane (1.5–5.1%)	Lithuania
Yang et al. [36]	Flavonoids (naringenin-7-O-*β*-d-glycoside, isoquercitrin, astragalin)	China
Lv et al. [37]	prenylated phthalide glycosides	China
Zhang et al. [38]	6,7-dimethoxy-4-hydroxy-1-naphthoic acid (1),(Z)-5-hydroxy-7-methoxy-4-[3-methyl-4-(O-*β*-d-xylopyranosyl)but-2-enyl]isobenzofuran-1(3H)-one (2).	China
Eshbakova and Aisa [39]	naringenin, helichrysum phthalide, diosmin, oleanolic acid	Republic of Uzbekistan
Morikawa et al. [40]	naringenin 7-O-*β*-D-glucopyranoside, apigenin 7-O-*β*-D-glucopyranoside, apigenin 7-O-gentiobioside, apigenin 7,4′-di-O-*β*-D-glucopyranoside	cultivated in Poland purchased Tochimoto Tenkaido Co., Ltd., Osaka, Japan
Albayrak et al. [41]	phenolic compounds	Turkey
Yong et al. [42]	*β*-sitosterol, stigmasterol, *β*-sitosterol, *β*-D-glucopyranoside, stigmasterol, caffeic acid ethyl ester.	China
Oji et al. [43]	limonene (21.2%), alpha-cadinol (18.2%), borneol (11.9%), delta-cadinene (9%), bornyl acetate (8%), alpha-humulene (7.3%).	Iran
Gradinaru et al. [44]	caffeic acid; flavonoids (apigenin, naringenin, apigenin-7-O-glucoside, naringenin-O-hexosides)	Romania
Moghadam et al. [45]	spathulenol (36.6%), *β*-pinene (12.5%)	Iran
Bandeira Reidel et al. [46]	*β*-caryophyllene (27–46%); (E)-2-hexenal; *β*-pinene (7.4%);	Italy
Bandeira Reidel et al. [47]	*β*-pinene (7.4%); *β*-caryophyllene (27.5%); *δ*-cadinene (3.2%); pentadecanoic acid, methyl ester (31%)	Italy
Babotă et al. [48]	phenolic compound; methoxylated flavone; sterolic compound;	Romania
Judzentiene et al. [49]	1,8-cineole (8.9%, one sample), *β*-caryophyllene (5.8–36.2%, 14 oils), *γ*- and *δ*-cadinene (5.8% and 9%); octadecane (7.1–22.3%).	Lithuania
Liu et al. [50]	linalool (2.81%); 4-acetyl-1-methylcyclohexene (1.88%); *β*-spathulenol (24.03%); caryophyllene oxide (3.05%); ledol (6.22%); hinesol (3.86%); *β*-eudesmol (2.56%); *α*-eudesmol (4.37%); *α*-cadinol (7.76%); *α*-bisabolol (5.71%)	Inner Mongolia, China
Stankov et al. [51]	oleic acid (30.28%), ethyl hexadecanoate (20.19%), linoleic acid (18.89%), sclareol (4.22%)	Turkey
Ivanović et al. [52]	phenolic compounds	Slovenia

**Table 2:** ANOVA *p*-values showing the significance of the differences among the three collections in terms of *H. arenarium* concentrations.

Constituent	*p*-Value	Constituent	*p*-Value	Constituent	*p*-Value
*α*-pinene	0.033 *	*β*-gurjunene	0.322	1-terpinen-4-ol	**0.002**
sabinene	0.716	germacrene D	**0.027**	long-chain alkane	**0.017**
*β*-pinene	**0.073**	germacra-4(15),5,10(14)-trien-1	**0.089**	n-tetradecane	0.138
D-limonene	0.403	monoterpenes	**0.004**	diterpenoids	0.355
trans-verbenol	0.400	sesquiterpenes	**0.084**		

* Significant effects that require multiple means comparison are shown in bold.

**Table 3:** Mean *H. arenarium* concentration (%) of *α*-pinene, *β*-pinene, 1-terpinen-4-ol, germacrene D, germacra-4(15),5,10(14)-trien, monoterpenes, sesquiterpenes, diterpenoids and long-chain alkane obtained from three collections (Location 1, Location 2 and Location 3) within the natural populations.

Collection	*α*-Pinene	*β*-Pinene	1-Terpinen-4-ol	Germacrene D	Germacra-4(15),5,10(14)-trien
689 Location 1	44.35 a *	2.85 a	0.92 c	3.56 b	1.14 ab
691 Location 2	36.60 b	2.30 b	2.11 a	5.33 a	1.09 b
699 Location 3	34.64 b	2.67 ab	1.26 b	4.83 a	1.37 a
**Collection**		Monoterpenes	Sesquiterpenes	Long-chain alkane	Diterpenoids
689 Location 1		73.99 a	16.08 b	4.33 b	3.25 b
691 Location 2		68.96 b	18.01 ab	6.23 a	3.45 ab
699 Location 3		65.72 c	19.41 a	5.69 a	4.27 a

* Within each constituent, means sharing the same letter are not significantly different.

**Table 4:** Overall mean *H. arenarium* concentration (%) of sabinene, D-limonene, trans-verbenol, n-tetradecane and *β*-gurjunene where there was no significant difference among the three collections.

Constituent	Overall Mean Concentration	$\sqrt{MSE}\;*$
Sabinene	10.80	0.607
D-limonene	2.11	0.151
trans-verbenol	3.18	0.217
n-tetradecane	2.36	0.135
*β*-gurjunene	3.61	0.259

* $\sqrt{MSE}$ = square root of the mean square error (*MSE*) that estimates the common standard deviation (*σ*).

**Table 5:** ANOVA *p*-values showing the significance of the differences among the three countries in terms of *H. italicum* concentrations.

Constituent	*p*-Value	Constituent	*p*-Value
*α*-Pinene	<0.001 *	*β*-Caryophyllene	**0.001**
D-Limonene	**0.019**	*p*-Cymen-7-ol acetate	**0.004**
2-Methyl butyl-2-methyl butyrate	**<0.001**	*α*-Guaiene	**<0.001**
Isoamyl tiglate	**0.004**	*γ*-Curcumene	**0.002**
1-Terpinen-4-ol	**0.001**	*β*-Himachalene	0.467
Nerol	**0.001**	*β*-Curcumene	0.232
Neryl acetate	**0.001**	Germacrene D-4-ol	**0.025**
*α*-Copaene	**0.049**	*γ*-Eudesmol	**0.027**
Italicene	**0.016**	tau.-Muurolol	0.751
*α*-cis-Bergamotene	**0.002**	*β*-Eudesmol	0.120
Sesquiterpenes	**0.048**	Monoterpenes	**<0.001**
Ester	0.669	Long-chain alkane	**0.069**

* Significant effects that require multiple means comparison are shown in bold.

**Table 6:** Mean *H. italicum* concentration (%) of *α*-pinene, D-limonene, 2-methyl butyl-2-methyl butyrate, isoamyl tiglate, 1-terpinen-4-ol, nerol, neryl acetate, *α*-copaene, italicene, *α*-cis-bergamotene, *β*-caryophyllene, p-cymen-7-ol acetate, *α*-guaiene, *γ*-curcumene, ger-macrene D-4-ol, *γ*-eudesmol, *β*-himachalene, monoterpenes, sesquiterpenes, ester and long-chain alkane obtained from three countries.

Country	*α*-Pinene	D-Limonene	2-Methyl butyl-2-methyl butyr	Isoamyl tiglate	1-Terpinen-4-ol	Nerol	Neryl acetate
Bosnia	13.74 a *	3.37 b	0.087 c	0.83 b	0.29 c	0.19 b	4.04 c
France	4.84 b	5.23 a	4.31 a	1.74 a	1.37 b	2.26 a	14.87 a
Corsica	2.83 c	4.94 a	3.44 b	1.93 a	1.67 a	2.50 a	12.37 b
Country	*α*-Copaene	Italicene	*α*-cis-Bergamotene	*β*-Caryophyllene	p-Cymen-7-ol acetate	*α*-Guaiene	*γ*-Curcumene
Bosnia	2.38 a	2.93 b	0.38 b	3.65 a	5.27 a	1.71 b	2.46 a
France	1.99 ab	4.67 a	1.19 a	0.38 b	2.47 b	3.98 a	0.93 b
Corsica	1.71 b	4.23 a	1.19 a	0.48 b	2.50 b	4.08 a	0.65 b
Country	Germacrene D-4-ol	*γ*-Eudesmol	*β*-Himachalene	Monoterpenes	Sesquiterpenes	Long-chain alkane	Ester
Bosnia	2.49 a	1.71 b	10.80 ab	29.83 c	54.80 a	0.74 b	7.84 ab
France	0.80 b	3.35 a	9.90 b	37.30 a	45.23 b	0.84 ab	7.96 a
Corsica	1.81 a	3.47 a	10.99 a	35.29 b	47.90 ab	0.99 a	7.09 c

* Within each constituent, means sharing the same letter are not significantly different.

**Table 7:** Overall mean *H. italicum* concentration (%) of *β*-Himachalene, *β*-Curcumene. tau.-Muurolol, *β*-Eudesmol, and Ester where there was no significant difference among the three collections.

Constituent	Overall Mean Concentration	$\sqrt{MSE}\;$
*β*-Himachalene	10.57	0.832
*β*-Curcumene	2.05	0.411
tau.-Muurolol	1.13	0.083
*β*-Eudesmol	1.44	0.208
Ester	7.63	0.982

$\sqrt{MSE}$ = square root of the mean square error (*MSE*) that estimates the common standard deviation (*σ*).

**Table 8:** Mean antimicrobial activities obtained from the three locations where *H. italicum* were collected.

Location	SA	EF	SP	PA	YE	SE	CA	CK	CT
Bosnia	9.33 b	4.00 b	8.33 b	2.33 b	2.33 b	5.67 a	5.33 a	4.67 b	5.67 a
Corsica	14.67 a	1.67 c	6.67 c	2.67 b	5.33 a	3.33 b	5.33 a	5.67 b	4.33 a
France	14.67 a	12.33 a	10.67 a	5.33 a	5.00 a	4.00 b	5.67 a	10.67 a	6.33 a

Within each constituent, means sharing the same letter are not significantly different. SA-*Staphylococcus aureus* subs. *aureus* CCM 4223, EF-*Enterococcus faecalis* CCM 4224, SP-*Streptococcus pneumonia* CCM 4501, PA-*Pseudomonas aeroginosa* CCM 1959, YE-*Yersinia enterocolitica* CCM 5671, SE-*Salmonella enterica* subsp. *enterica* CCM 3807, CA-*Candida albicans* CCM 8186, CK-*C. krusei* CCM 8271 and CT-*C. tropicalis* CCM 8223 (CT).

## Data Availability

Data are contained within the article.

## Supplementary Materials

The following are available online at https://www.mdpi.com/article/10.3390/plants11070951/s1, Table S1: Constituents and concentrations of *Helichrysum arenarium* from Bulgaria. The min-max range represents the *H. arenarium* essential oil constituents from the three locations in Bulgaria. Table S2: Constituents and concentrations of *Helichrysum italicum* introduced from France, Corsica and Bosnia. The min-max range includes variation in concentrations of the essential oil constituents from all three locations; France, Corsica, and Bosnia.
