# Supplementary material for: Chemical Profile and Antimicrobial Activity of the Essential Oils of Helichrysum arenarium (L.) Moench. and Helichrysum italicum (Roth.) G. Don
Source: Plants (Basel). 2022 Mar 31;11(7):951. doi: 10.3390/plants11070951 (PMC9002512; doi:10.3390/plants11070951)
Supplement: Supplementary file 1 [file plants-11-00951-s001.zip › plants-1603507-SI.pdf]

Supplemental Table S1. Constituents and concentrations of *Helichrysum arenarium* from Bulgaria. The min-max range represents the *H. arenarium* essential oil constituents from the three locations in Bulgaria.

**№**	**Compounds**	**RI**	**min-max**
1	Tricyclene	920	0.096-0.157
2	α-Thujene	924	0.048-0.300
3	α-Pinene	932	32.713-45.655
4	Camphene	946	0.252-1.373
5	Thuja-2,4(10)-diene	953	0.263-1.118
6	Sabinene	969	10.044-11.357
7	β-Pinene	974	2.247-3.019
8	α-Phellandrene	1002	0.272-0.324
9	α-Terpinene	1015	0.193-0.523
10	p-Cymene	1020	0.271-0.425
11	D-Limonene	1023	1.942-2.356
12	γ-Terpinene	1055	0.165-1.008
13	(2E)-Octen-1-ol	1062	0.330-0.549
14	Terpinolene	1085	0.259-0.561
15	6-Camphenone	1094	0.066-0.409
16	α-Pinene oxide	1098	0.233-0.367
17	n-Unecane	1100	0.188-0.324
18	Thujone	1102	0.413-0.468
19	6-Camphenol	1111	0.363-1.605
20	α-Campholenal	1123	0.325-0.979
21	cis-Verbenol	1136	0.881-1.014

22	trans-Verbenol	1140	2.868-3.454
23	Pinocarvone	1160	0.169-0.200
24	Mentha-1,5-dien-8-ol	1167	0.355-0.609
25	1-Terpinen-4-ol	1178	0.862-2.059
26	p-Cymen-8-ol	1180	0.135-0.207
27	2-Pinen-10-ol	1194	0.640-0.772
28	Verbenone	1205	0.407-0.685
29	trans-Carveol	1216	0.263-0.344
30	Carvone	1239	0.160-0.241
31	Bornyl acetate	1255	0.786-1.347
32	α-Longipinene	1348	0.121-0.216
33	α-Copaene	1374	0.074-0.124
34	β-Elemene	1389	0.151-0.192
35	n-Tetradecane	1400	2.077-2.574
36	Methyleugenol	1405	0.065-0.284
37	β-Caryophyllene	1418	0.540-0.684
38	Carvone hydrate	1423	0.086-0.111
39	β-Copaene	1429	0.00-0.354
40	β-Gurjunene	1432	3.256-3.980
41	α-Humulene	1451	0.213-0.335
42	Germacrene D	1484	3.294-5.546
43	n-Pentadecane	1500	0.369-0.431
44	Germacrene D-4-ol	1572	0.200-0.291
45	(-)-Spathulenol	1577	0.249-0.717

46	Caryophyllene oxide	1582	0.345-0.589
47	γ-Eudesmol	1630	0.146-0.376
48	tau.-Cadinol	1638	0.665-0.923
49	α-Muurolol	1646	0.828-1.735
50	β-Eudesmol	1650	0.418-0.857
51	neo-Intermedeol	1657	0.279-0.342
52	Intermedeol	1665	0.663-1.075
53	Germacra-4(15),5,10(14)-trien-1α-ol	1686	1.063-1.450
54	n-Heptadecane	1700	0.317-0.449
55	Curcumenol	1732	0.152-0.209
56	Aristolone	1761	0.735-1.205
57	14-hydroxy-α-Muurolene	1778	0.49-0.763
58	n-Octadecane	1800	0.088-0.412
59	Hexahydrofarnesyl acetone	1833	0.175-0.520
60	n-Nonadecane	1900	0.115-0.159
61	Manoyl oxide	1994	0.099-0.365
62	n-Eicosane	2000	0.292-0.925
63	n-Heneicosane	2100	0.205-0.377
64	Laurenan-2-one	2113	0.101-0.313
65	Methyl octadecanoate	2124	0.190-0.291
66	Linoleic acid	2133	0.00-0.202
67	Oleic acid	2142	0.00-0.134
68	Grandiflorene	2175	0.00-0.119
69	n-Docosane	2200	0.109-0.715

70	Phyllocladanol	2209	0.404-2.356
71	(E)-Phytol acetate	2218	0.874-1.332
72	Sclareol	2225	0.564-0.708
73	7α-hydroxy-Manool	2237	0.155-0.229
74	3α-hydroxy-Manool	2293	0.240-0.946
75	n-Tricosane	2300	0.365-0.574

Supplemental Table S2. Constituents and concentrations of *Helichrysum italicum* introduced from France, Corsica and Bosnia. The min-max range includes variation in concentrations of the essential oil constituents from all three locations; France, Corsica, and Bosnia.

**№**	**Compounds**	**RI**	**min-max of the three origins**
**1**	4-methyl-2-Pentanol	745	0.00-0.513
**2**	2-Methyloctane	861	0.00-0.803
**3**	n-Nonane	900	0.089-0.237
**4**	α-Pinene	932	2.541-14.268
**5**	Camphene	946	0.736-1.019
**6**	Sabinene	969	0.087-0.322
**7**	β-Pinene	974	0.703-2.117
**8**	α-Phellandrene	1002	0.165-0.223
**9**	α-Terpinene	1015	0.133-0.411
**10**	p-Cymene	1020	0.247-0.762
**11**	D-Limonene	1023	3.118-5.483
**12**	Eucalyptol	1025	0.317-0.989
**13**	β-trans-Ocimene	1040	0.185-0.558
**14**	Isobutyl angelate	1044	0.239-0.552
**15**	γ-Terpinene	1055	0.316-0.924
**16**	Terpinolene	1085	0.194-0.373
**17**	2-Nonanone	1089	0.120-0.767
**18**	β-Linalool	1096	0.098-0.923
**19**	2-Methyl butyl-2-methyl butyrate	1123	0.081-4.357
**20**	Isoamyl angelate	1143	0.094-0.199

**21**	Isoamyl tiglate	1149	0.777-2.057
**22**	1-Terpinen-4-ol	1178	0.272-1.776
**23**	(2Z)-Octenol acetate	1197	0.153-0.750
**24**	n-Decanal	1203	0.080-0.654
**25**	Nerol	1226	0.178-2.661
**26**	tetrahydro-Linalyl acetate	1232	0.062-0.608
**27**	3-Methyl pentyl angelate	1249	0.097-0.494
**28**	2-Undecanone	1291	0.079-0.413
**29**	n-Tridecane	1300	0.089-0.738
**30**	Neryl acetate	1359	3.486-15.105
**31**	(2E)-Undecenol	1266	0.110-0.991
**32**	α-Copaene	1373	1.598-2.534
**33**	n-Tetradecane	1400	0.055-0.152
**34**	Italicene	1406	2.739-4.738
**35**	α-cis-Bergamotene	1411	0.354-1.272
**36**	β-Caryophyllene	1417	0.377-3.888
**37**	p-Cymen-7-ol acetate	1421	2.343-5.602
**38**	Linalool butanoate	1423	0.226-0.391
**39**	β-Copaene	1429	0.153-1.219
**40**	α-Guaiene	1436	1.603-4.154
**41**	(Z)-β-Farnesene	1439	0.451-0.626
**42**	(2Z,6E)-Dodecadien-1-al	1444	0.155-1.137
**43**	α-Himachalene	1450	0.328-0.686
**44**	n-Dodecanol	1470	0.561-0.797
**45**	Undecanal, dimethyl acetal	1475	0.550-0.851
**46**	γ-Curcumene	1481	0.612-2.613
**47**	β-Himachalene	1500	9.744-11.705
**48**	β-Curcumene	1513	1.359-2.611
**49**	δ-Cadinene	1522	0.557-5.631
**50**	α-Cadinene	1537	0.403-3.554
**51**	α-Calacorene	1544	0.561-1.732
**52**	cis-Cadinene ether	1550	0.841-1.147
**53**	trans-Cadinene ether	1556	0.404-0.922
**54**	β-Calacorene	1560	0.386-0.989
**55**	Germacrene D-4-ol	1574	0.790-2.801
**56**	Pentyl salicylate	1576	0.239-1.548
**57**	(-)-Spathulenol	1578	0.631-0.910
**58**	Caryophyllene oxide	1582	0.085-0.522
**59**	Globulol	1590	0.598-3.061

**60**	Guaiol	1600	0.068-1.016
**61**	Junenol	1618	0.00-0.898
**62**	γ-Eudesmol	1630	1.345-3.713
**63**	tau.-Cadinol	1638	0.230-0.879
**64**	tau.-Muurolol	1642	1.071-1.229
**65**	β-Eudesmol	1650	1.006-1.837
**66**	α-Eudesmol	1650	0.698-3.693
**67**	Geranyl valerate	1656	0.252-0.921
**68**	(6Z)-Pentadecen-2-one	1667	0.285-0.861
**69**	Germacra-4(15),5,10(14)-trien-1α-ol	1686	0.299-0.562
**70**	n-Heptadecane	1700	0.092-0.137
**71**	(2Z,6E)-Farnesol	1722	0.200-0.709
**72**	(2E,6E)-Farnesal	1740	0.090-0.159
**73**	14-oxy-α-Muurolene	1767	0.306-0.934
**74**	14-hydroxy-α-Muurolene	1778	0.251-0.449
**75**	(2E,6E)-Methyl farnesoate	1784	0.549-1.185
**76**	1-Octadecene	1791	0.36-0.938
**77**	n-Octadecane	1800	0.00-0.126
**78**	Methyl octadecanoate	2124	0.00-0.158
**79**	n-Docosane	2200	0.00-0.119
